# Supplementary material for: Assessment of Phenotypic Variations and Correlation among Seed Composition Traits in Mutagenized Soybean Populations
Source: Genes (Basel). 2019 Nov 27;10(12):975. doi: 10.3390/genes10120975 (PMC6947669; doi:10.3390/genes10120975)
Supplement: Supplementary file 1 [file genes-10-00975-s001.pptx]

## Slide 1
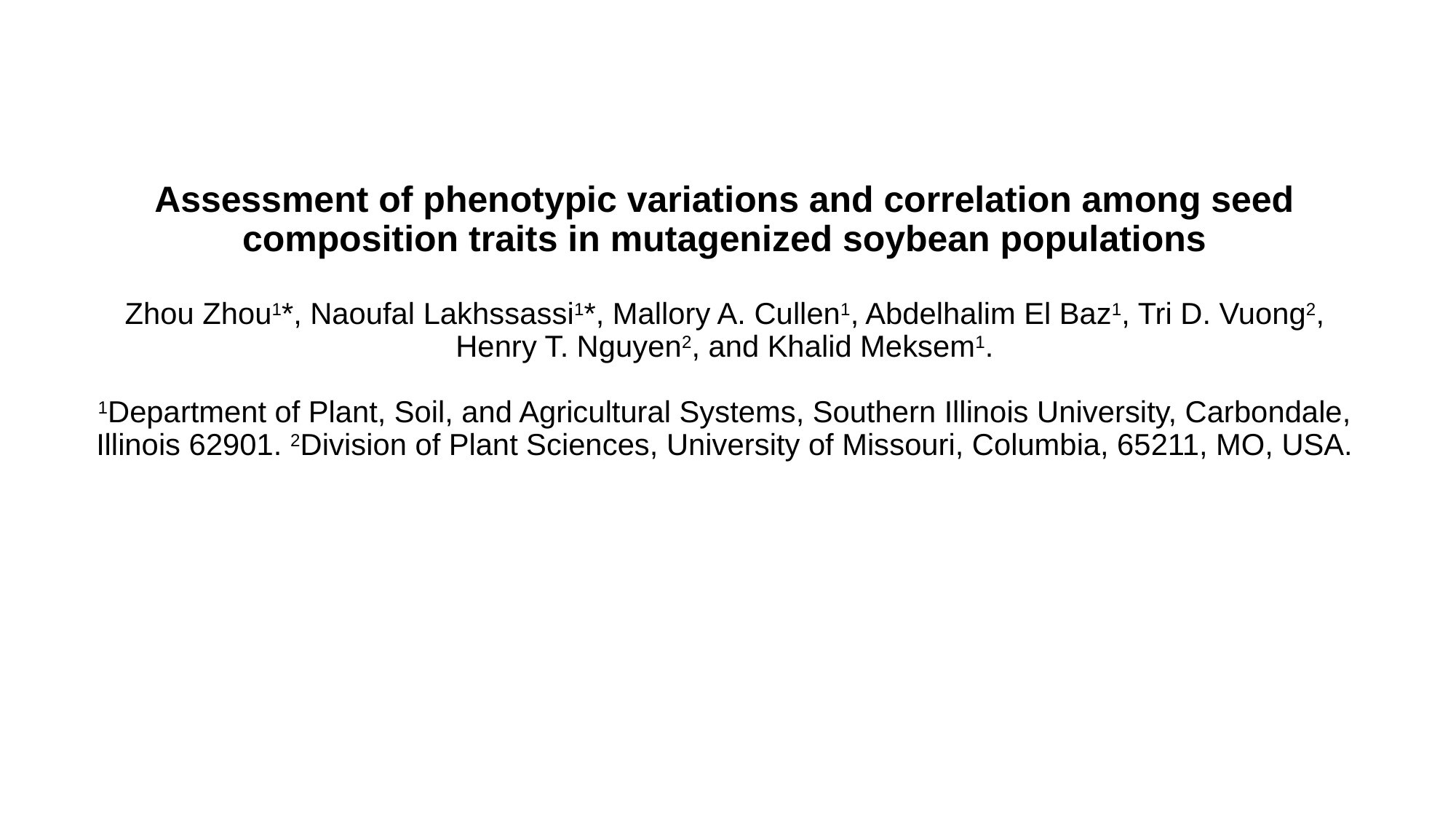

# Assessment of phenotypic variations and correlation among seed composition traits in mutagenized soybean populations Zhou Zhou1*, Naoufal Lakhssassi1*, Mallory A. Cullen1, Abdelhalim El Baz1, Tri D. Vuong2, Henry T. Nguyen2, and Khalid Meksem1. 1Department of Plant, Soil, and Agricultural Systems, Southern Illinois University, Carbondale, Illinois 62901. 2Division of Plant Sciences, University of Missouri, Columbia, 65211, MO, USA.

## Slide 2
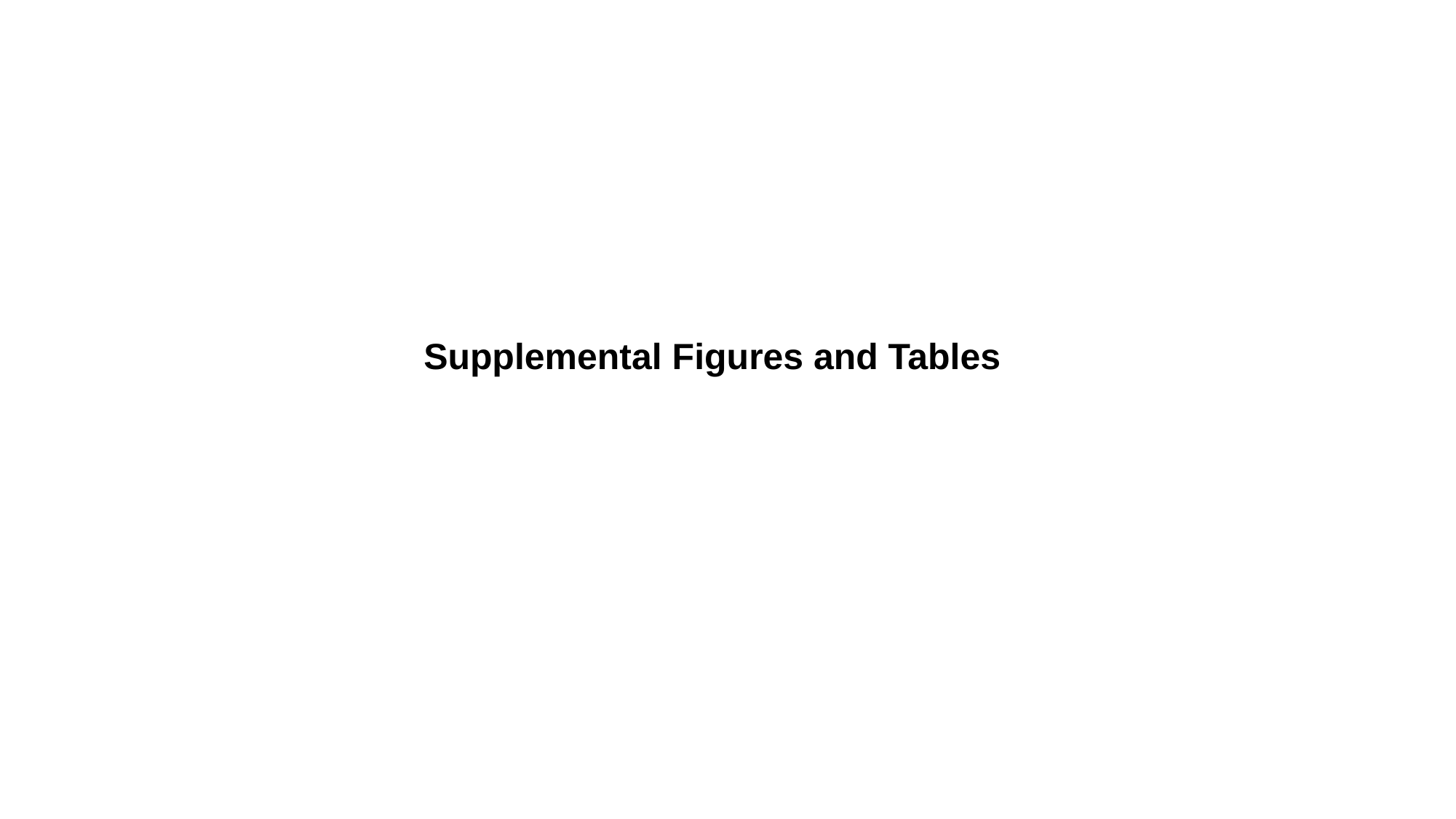

Supplemental Figures and Tables

## Slide 3
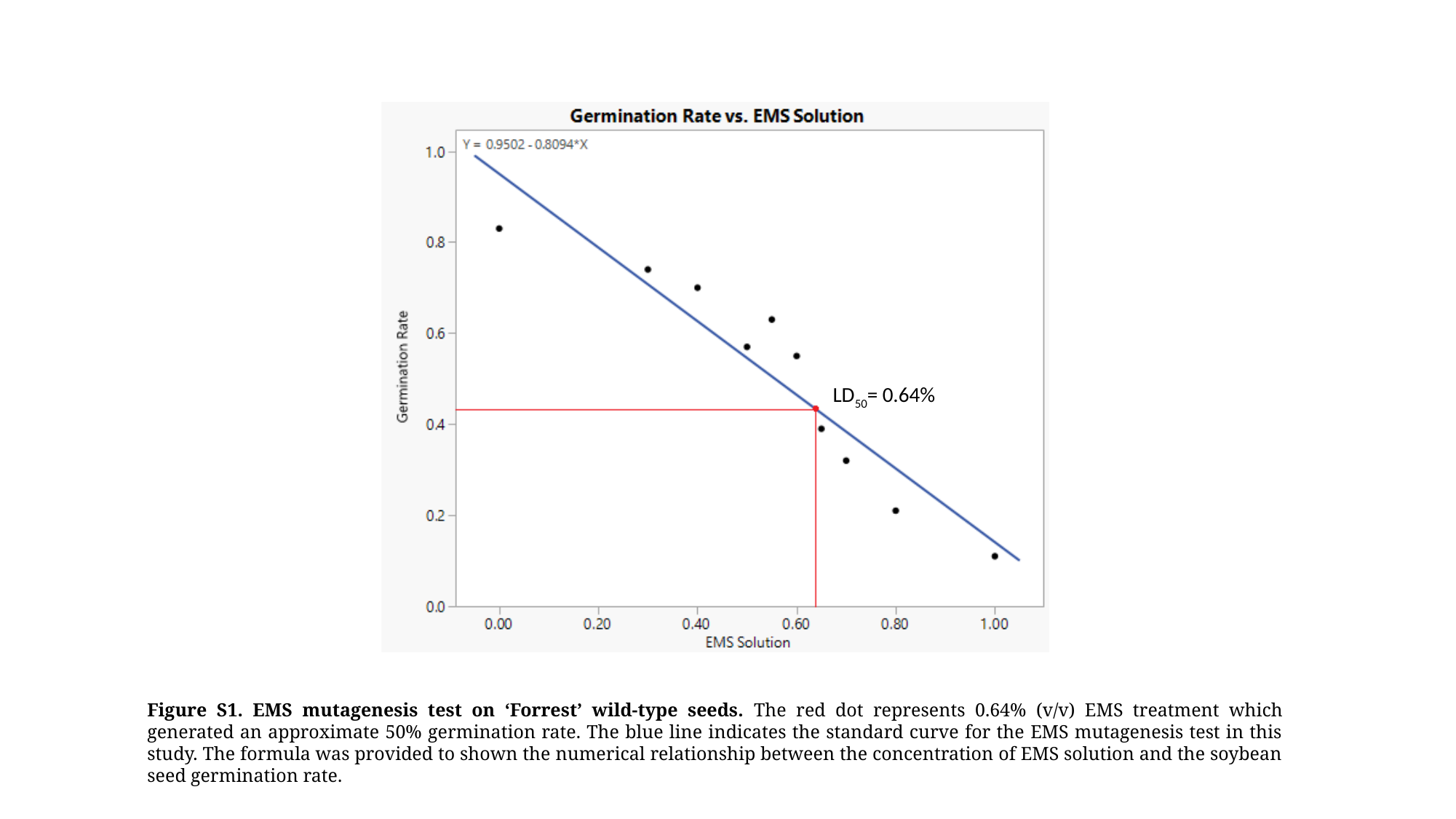

LD50= 0.64%
Figure S1. EMS mutagenesis test on ‘Forrest’ wild-type seeds. The red dot represents 0.64% (v/v) EMS treatment which generated an approximate 50% germination rate. The blue line indicates the standard curve for the EMS mutagenesis test in this study. The formula was provided to shown the numerical relationship between the concentration of EMS solution and the soybean seed germination rate.

## Slide 4
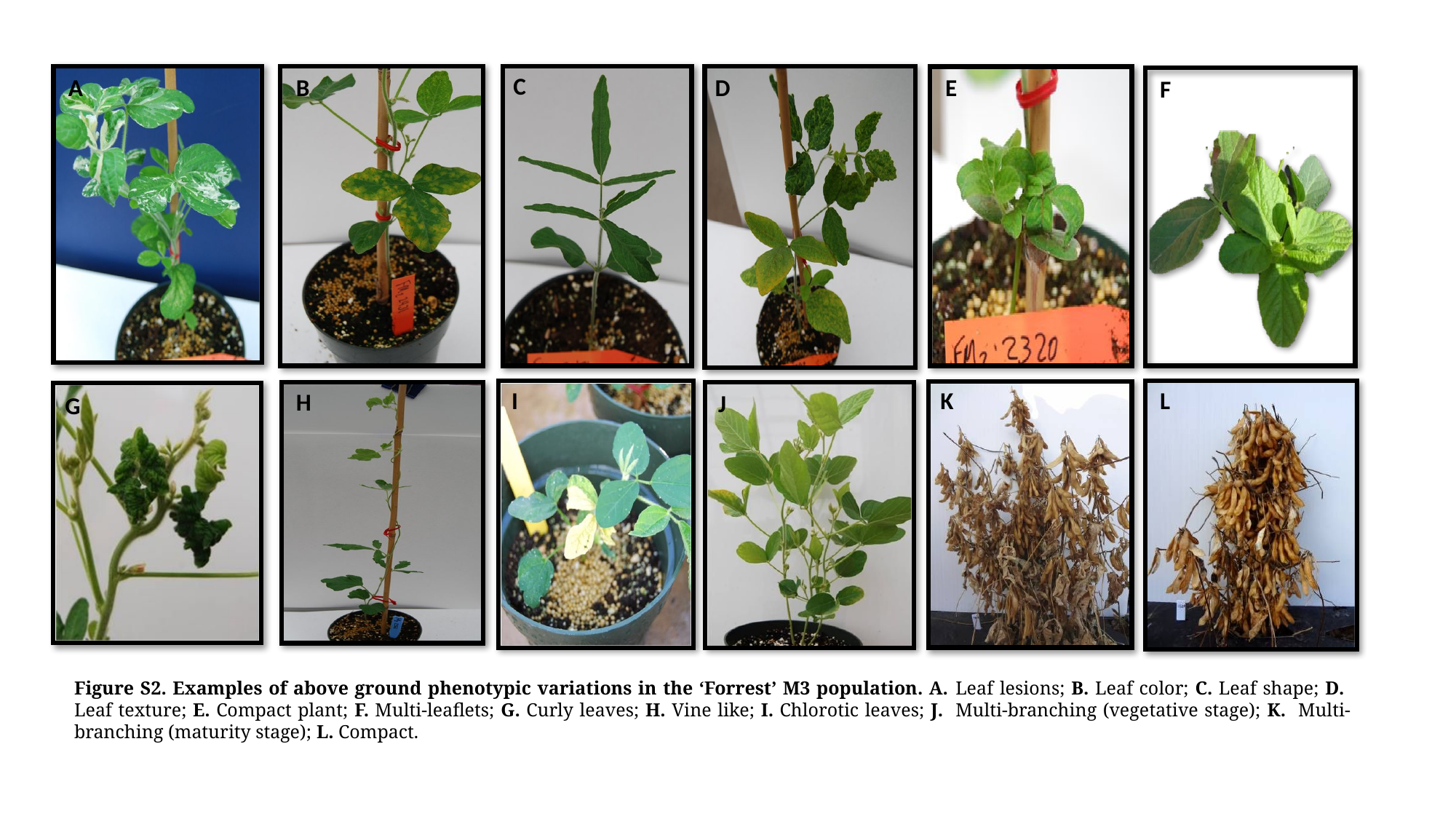

C
B
D
E
A
F
L
I
K
H
J
G
Figure S2. Examples of above ground phenotypic variations in the ‘Forrest’ M3 population. A. Leaf lesions; B. Leaf color; C. Leaf shape; D. Leaf texture; E. Compact plant; F. Multi-leaflets; G. Curly leaves; H. Vine like; I. Chlorotic leaves; J. Multi-branching (vegetative stage); K. Multi-branching (maturity stage); L. Compact.

## Slide 5
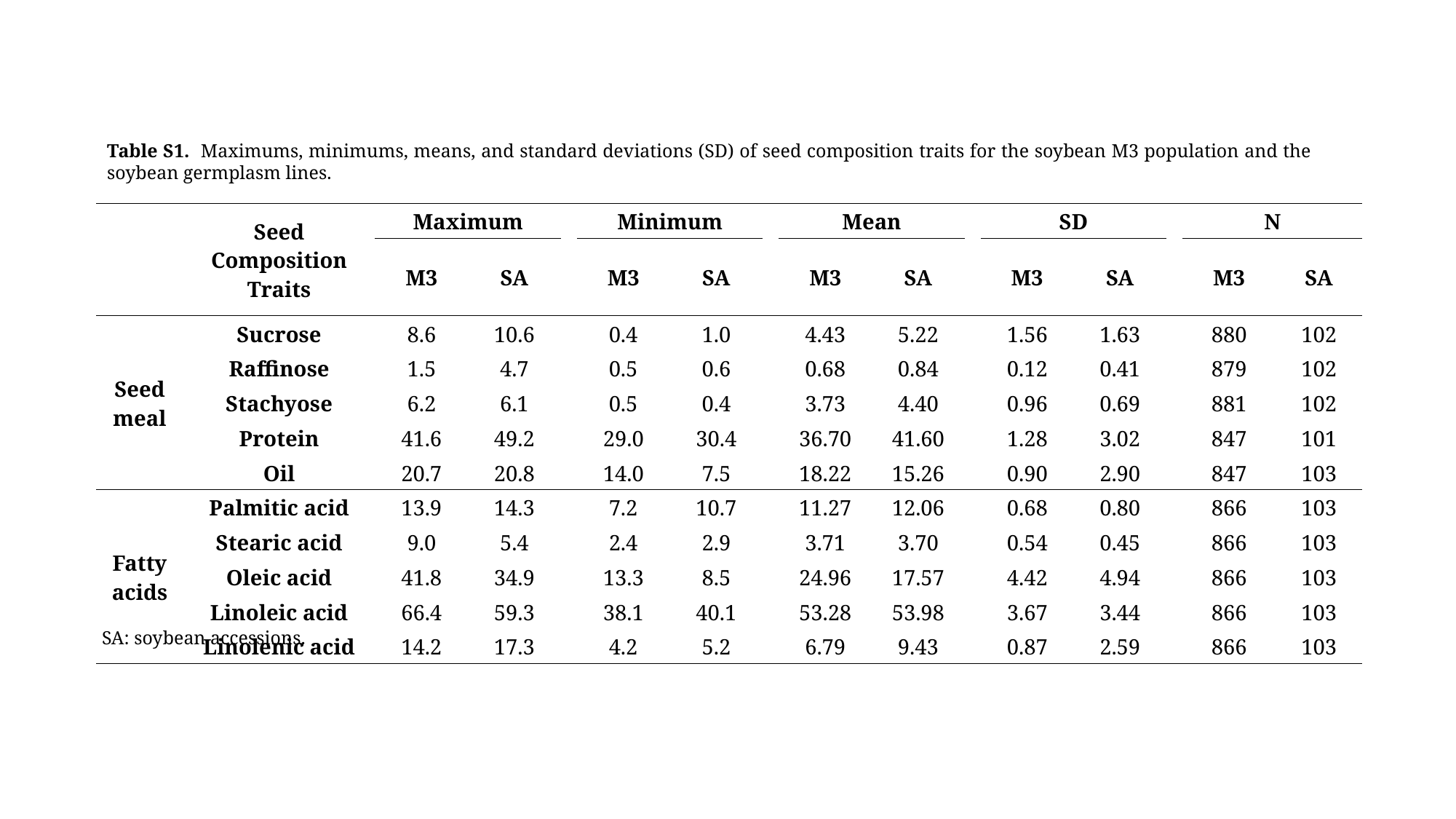

Table S1. Maximums, minimums, means, and standard deviations (SD) of seed composition traits for the soybean M3 population and the soybean germplasm lines.
| | Seed Composition Traits | Maximum | | | Minimum | | | Mean | | | SD | | | N | |
| --- | --- | --- | --- | --- | --- | --- | --- | --- | --- | --- | --- | --- | --- | --- | --- |
| | | M3 | SA | | M3 | SA | | M3 | SA | | M3 | SA | | M3 | SA |
| Seed meal | Sucrose | 8.6 | 10.6 | | 0.4 | 1.0 | | 4.43 | 5.22 | | 1.56 | 1.63 | | 880 | 102 |
| | Raffinose | 1.5 | 4.7 | | 0.5 | 0.6 | | 0.68 | 0.84 | | 0.12 | 0.41 | | 879 | 102 |
| | Stachyose | 6.2 | 6.1 | | 0.5 | 0.4 | | 3.73 | 4.40 | | 0.96 | 0.69 | | 881 | 102 |
| | Protein | 41.6 | 49.2 | | 29.0 | 30.4 | | 36.70 | 41.60 | | 1.28 | 3.02 | | 847 | 101 |
| | Oil | 20.7 | 20.8 | | 14.0 | 7.5 | | 18.22 | 15.26 | | 0.90 | 2.90 | | 847 | 103 |
| Fatty acids | Palmitic acid | 13.9 | 14.3 | | 7.2 | 10.7 | | 11.27 | 12.06 | | 0.68 | 0.80 | | 866 | 103 |
| | Stearic acid | 9.0 | 5.4 | | 2.4 | 2.9 | | 3.71 | 3.70 | | 0.54 | 0.45 | | 866 | 103 |
| | Oleic acid | 41.8 | 34.9 | | 13.3 | 8.5 | | 24.96 | 17.57 | | 4.42 | 4.94 | | 866 | 103 |
| | Linoleic acid | 66.4 | 59.3 | | 38.1 | 40.1 | | 53.28 | 53.98 | | 3.67 | 3.44 | | 866 | 103 |
| | Linolenic acid | 14.2 | 17.3 | | 4.2 | 5.2 | | 6.79 | 9.43 | | 0.87 | 2.59 | | 866 | 103 |
SA: soybean accessions.

## Slide 6
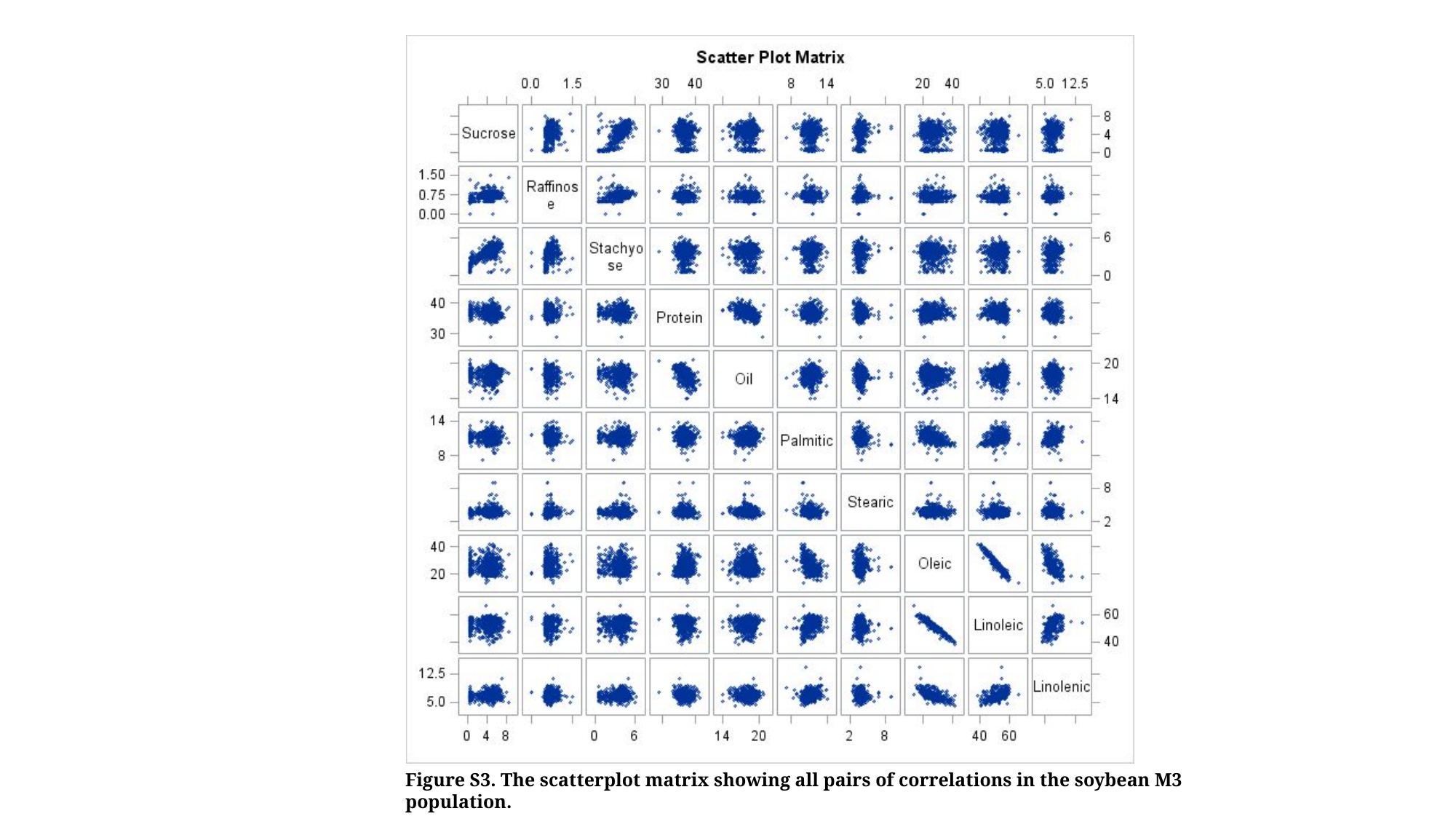

Figure S3. The scatterplot matrix showing all pairs of correlations in the soybean M3 population.

## Slide 7
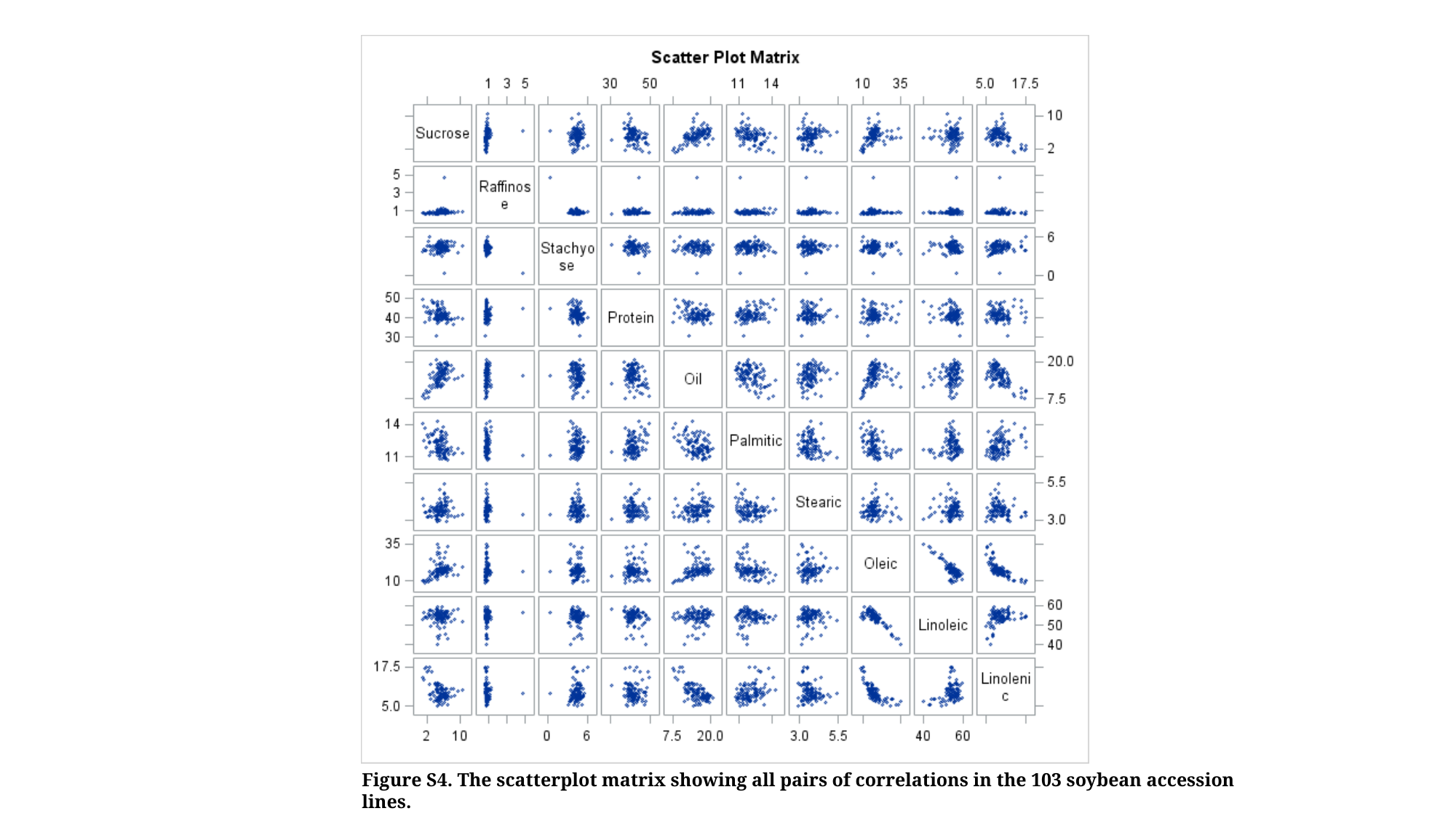

Figure S4. The scatterplot matrix showing all pairs of correlations in the 103 soybean accession lines.

## Slide 8
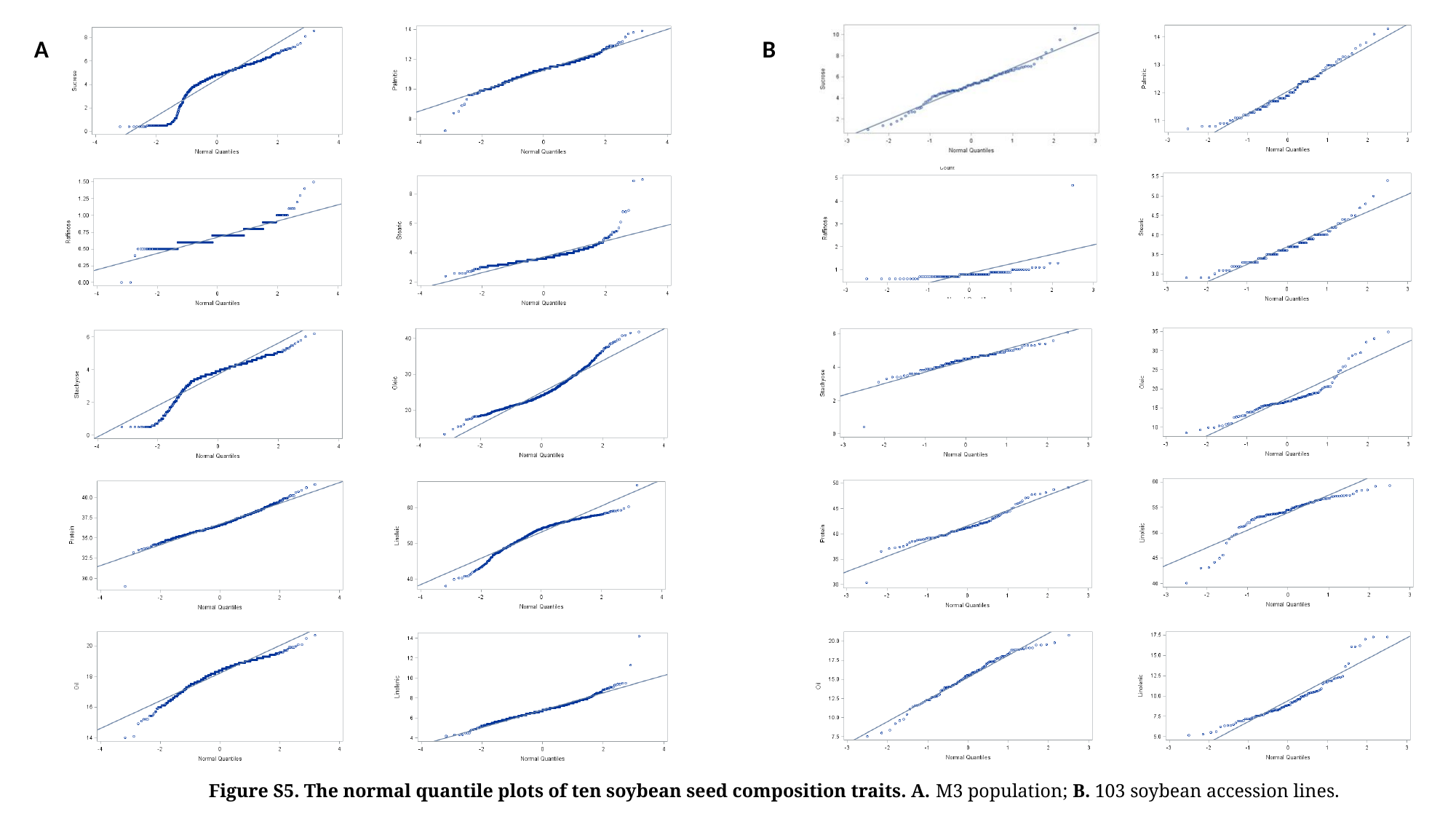

A
B
Figure S5. The normal quantile plots of ten soybean seed composition traits. A. M3 population; B. 103 soybean accession lines.

## Slide 9
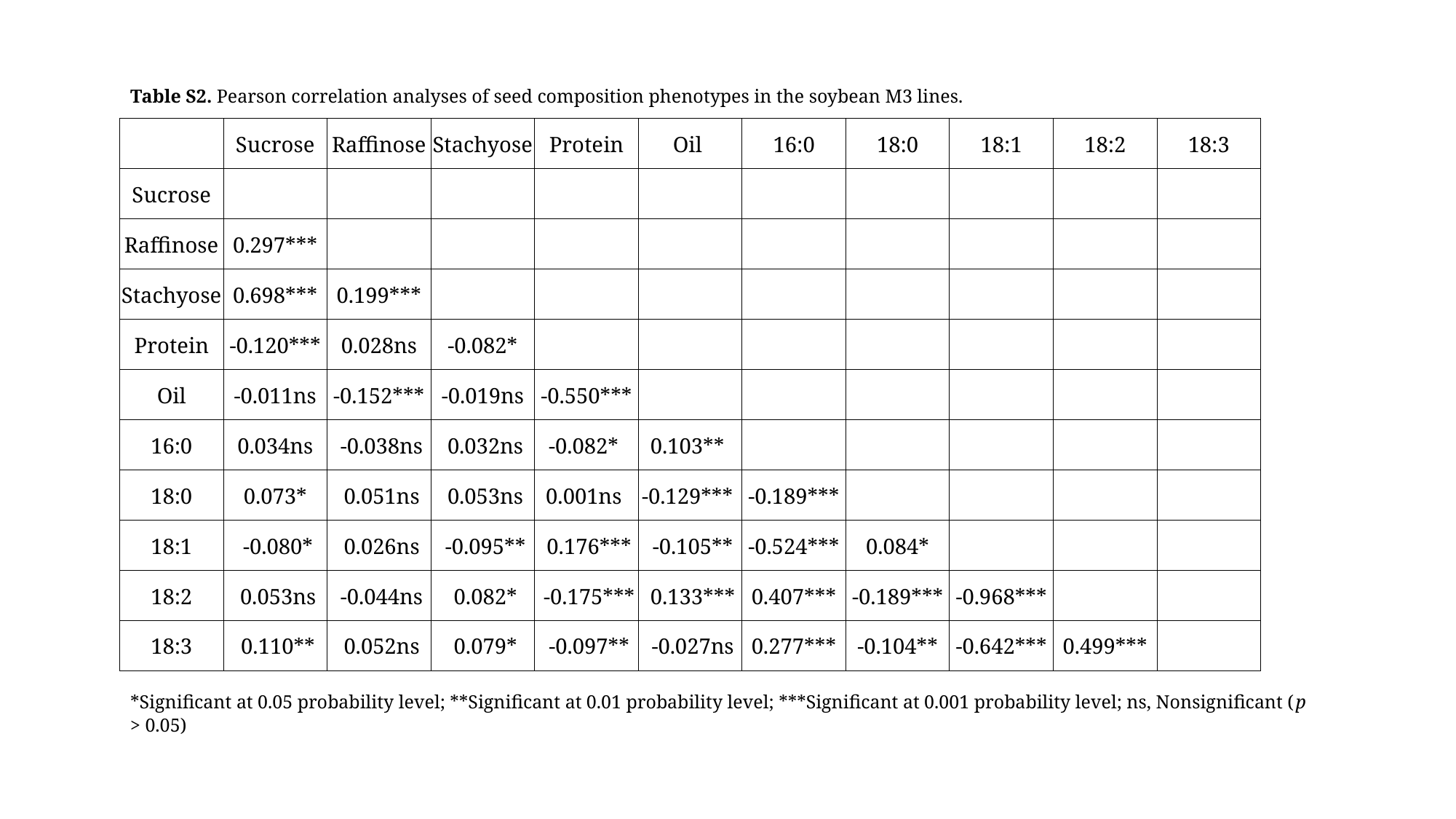

Table S2. Pearson correlation analyses of seed composition phenotypes in the soybean M3 lines.
| | Sucrose | Raffinose | Stachyose | Protein | Oil | 16:0 | 18:0 | 18:1 | 18:2 | 18:3 |
| --- | --- | --- | --- | --- | --- | --- | --- | --- | --- | --- |
| Sucrose | | | | | | | | | | |
| Raffinose | 0.297\*\*\* | | | | | | | | | |
| Stachyose | 0.698\*\*\* | 0.199\*\*\* | | | | | | | | |
| Protein | -0.120\*\*\* | 0.028ns | -0.082\* | | | | | | | |
| Oil | -0.011ns | -0.152\*\*\* | -0.019ns | -0.550\*\*\* | | | | | | |
| 16:0 | 0.034ns | -0.038ns | 0.032ns | -0.082\* | 0.103\*\* | | | | | |
| 18:0 | 0.073\* | 0.051ns | 0.053ns | 0.001ns | -0.129\*\*\* | -0.189\*\*\* | | | | |
| 18:1 | -0.080\* | 0.026ns | -0.095\*\* | 0.176\*\*\* | -0.105\*\* | -0.524\*\*\* | 0.084\* | | | |
| 18:2 | 0.053ns | -0.044ns | 0.082\* | -0.175\*\*\* | 0.133\*\*\* | 0.407\*\*\* | -0.189\*\*\* | -0.968\*\*\* | | |
| 18:3 | 0.110\*\* | 0.052ns | 0.079\* | -0.097\*\* | -0.027ns | 0.277\*\*\* | -0.104\*\* | -0.642\*\*\* | 0.499\*\*\* | |
*Significant at 0.05 probability level; **Significant at 0.01 probability level; ***Significant at 0.001 probability level; ns, Nonsignificant (p > 0.05)

## Slide 10
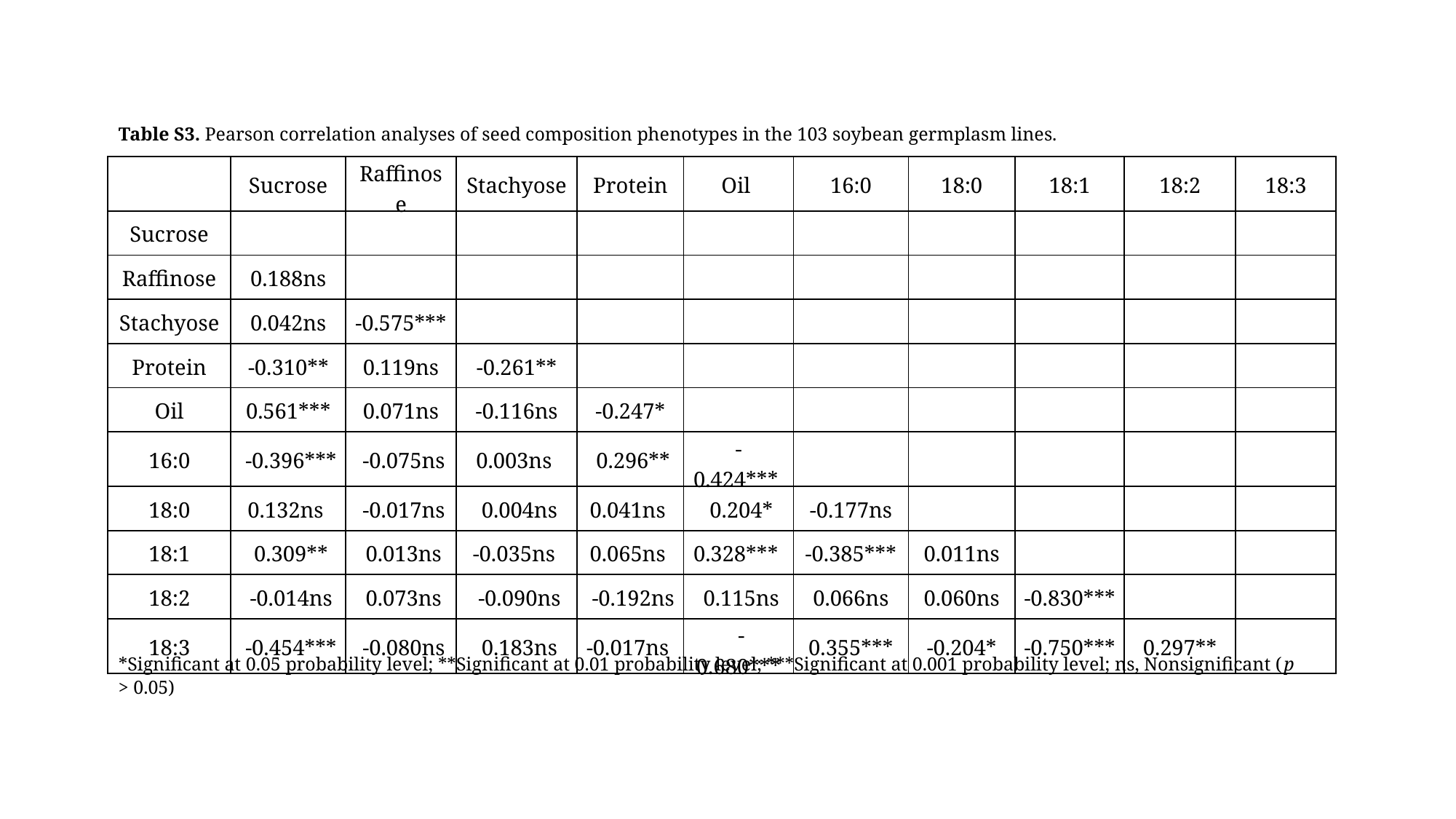

Table S3. Pearson correlation analyses of seed composition phenotypes in the 103 soybean germplasm lines.
| | Sucrose | Raffinose | Stachyose | Protein | Oil | 16:0 | 18:0 | 18:1 | 18:2 | 18:3 |
| --- | --- | --- | --- | --- | --- | --- | --- | --- | --- | --- |
| Sucrose | | | | | | | | | | |
| Raffinose | 0.188ns | | | | | | | | | |
| Stachyose | 0.042ns | -0.575\*\*\* | | | | | | | | |
| Protein | -0.310\*\* | 0.119ns | -0.261\*\* | | | | | | | |
| Oil | 0.561\*\*\* | 0.071ns | -0.116ns | -0.247\* | | | | | | |
| 16:0 | -0.396\*\*\* | -0.075ns | 0.003ns | 0.296\*\* | -0.424\*\*\* | | | | | |
| 18:0 | 0.132ns | -0.017ns | 0.004ns | 0.041ns | 0.204\* | -0.177ns | | | | |
| 18:1 | 0.309\*\* | 0.013ns | -0.035ns | 0.065ns | 0.328\*\*\* | -0.385\*\*\* | 0.011ns | | | |
| 18:2 | -0.014ns | 0.073ns | -0.090ns | -0.192ns | 0.115ns | 0.066ns | 0.060ns | -0.830\*\*\* | | |
| 18:3 | -0.454\*\*\* | -0.080ns | 0.183ns | -0.017ns | -0.680\*\*\* | 0.355\*\*\* | -0.204\* | -0.750\*\*\* | 0.297\*\* | |
*Significant at 0.05 probability level; **Significant at 0.01 probability level; ***Significant at 0.001 probability level; ns, Nonsignificant (p > 0.05)
